# Western Pacific regional engagement  in the Emergency Medical Team Global Meeting 2024

**DOI:** 10.5365/wpsar.2025.16.2.1261

**Published:** 2025-05-24

**Authors:** Erin Noste, Anthony Cook, Jan-Erik Larsen, Pierre-Yves Beauchemin, Vannda Kab, Dulamragchaa Buyanbaatar, Eystein Grusd, Sabrina Angela Tayo, Sean T Casey

**Affiliations:** a World Health Organization Western Pacific Regional Office, Manila, Philippines.; b Department of Emergency Medicine, University of California San Diego, San Diego, California, United States of America.; c World Health Organization Representative Office for Cambodia, Phnom Penh, Cambodia.; d World Health Organization Representative Office for Mongolia, Ulaanbaatar, Mongolia.; e School of Population Health, Faculty of Medicine and Health, University of New South Wales, Sydney, New South Wales, Australia.

The 6th Emergency Medical Team (EMT) Global Meeting was held in Abu Dhabi, United Arab Emirates on 5–7 November 2024. (*1*) This was the largest EMT Global Meeting to date, with over 1300 participants from more than 140 countries across all six regions of the World Health Organization (WHO). Discussions focused on health emergency response, coordination, lessons identified, best practices and future advancements in EMTs. The WHO Western Pacific Region was the second most represented region attending the meeting, with over 100 participants from 23 of the 37 countries and areas in the Region. The Western Pacific is home to 16 WHO-classified EMTs, and almost every country or area in the Region has a national and/or international EMT. The meeting provided an opportunity for participants to share, collaborate with and learn from colleagues both regionally and globally.

Participants and representatives from the Region actively contributed to various panels, discussions, working sessions and abstract presentations during the 3-day meeting. The EMT Global Meeting’s core theme was the implementation of the EMT 2030 strategy, expanding on the minimum standards set in the *Classification and minimum standards for emergency medical teams*. (*2*, *3*) A half-day Western Pacific Regional Meeting was held on the first day to bring together EMT members from the Region to celebrate successes, share lessons identified and plan for future nationally led, regionally supported health emergency responses.

The Western Pacific Region was well represented throughout the EMT Global Meeting (**Table 1**). EMT members from the Region presented in almost every plenary, technical and research-focused session (**Table 2**). Over 200 abstracts were submitted from 58 different countries for presentation. Of the 32 oral abstracts accepted for presentation, 13 were from the Region (**Table 2**), while 22 additional research abstracts were accepted for poster/digital presentation from the Region. Mercy Malaysia, Singapore Ministry of Health EMT and three teams from the Philippine Emergency Medical Assistance Team were recognized as newly classified international EMTs. The Australian Medical Assistance Team, New Zealand Medical Assistance Team and Japan Disaster Relief Team were recognized for their reclassification.

**Table 1:** Participation of representatives from countries/territories/areas in the WHO Western Pacific Region at the Emergency Medical Team Global Meeting, Abu Dhabi, United Arab Emirates, 5–7 November 2024

Country/territory/area	No. of participants
**American Samoa (USA)**	**1**
**Australia**	**14**
**Cambodia**	**4**
**China**	**6**
**Cook Islands**	**2**
**Fiji**	**3**
**Japan**	**12**
**Kiribati**	**1**
**Lao People's Democratic Republic**	**2**
**Malaysia**	**2**
**Mongolia**	**3**
**New Zealand**	**6**
**Northern Mariana Islands, Commonwealth of the (USA)**	**2**
**Palau**	**3**
**Papua New Guinea**	**3**
**Philippines**	**14**
**Republic of Korea**	**5**
**Samoa**	**4**
**Singapore**	**2**
**Solomon Islands**	**3**
**Tonga**	**2**
**Vanuatu**	**2**
**Viet Nam**	**1**
**WHO Western Pacific Regional Office**	**8**
**Total**	**105**

WHO: World Health Organization.

**Table 2:** Topics presented by representatives of countries/territories/areas in the WHO Western Pacific Region at the WHO Emergency Medical Team Global Meeting, Abu Dhabi, 5–7 November 2024

Country/territory/area	Topic
**Australia**	**Keynote speech** **Expanding partnerships** **All-hazards, One Health** **Concrete challenges from EMT operations and particularities of medical civil-military coordination** **New challenges: modularization and implementation strategies** **Towards a One EMT information management system – innovative online toolbox for AUSMAT and EMTs: enhancing information sharing and operational transparency** **Review capacity and standards of local EMTs in the post-pandemic period** **AUSMAT rehabilitation team member training** **Evaluation of the AUSMAT mentorship pilot program** **The characteristics of high-performance teams for infectious disease responses: an AUSMAT team leader's perspective** **Ready, set, deploy: AUSMAT’s surgical cache gets a makeover** **Evaluating and enhancing team member training: an 8-week program for AUSMAT using modern collaboration techniques and adult learning principles**
**Fiji**	**All about quality – a local EMT deployment to Fiji** **New challenges: modularization and implementation strategies** **FEMAT response to Tropical Cyclone Cody** **FEMAT response to leptospirosis outbreak in Navosa, Fiji** **FEMAT – Tuvalu COVID-19 surge support**
**Japan**	**Enhancing emergency response** **Towards a One EMT information management system** **Joint operation among EMT, FETP and PHRRT during Noto earthquake Japan 2024** **Japan disaster relief medical mission operating system – key achievement and lessons learned** **Health checkup of EMT members during 2024 Noto peninsula earthquake in Japan** **Innovative occupational health system for EMT staff implemented by the EMTCC during Noto earthquake 2024 in Japan** **Assessment of the quality of MDS data collected by EMTs during Idai Cyclone of Mozambique** **Relationship between fatigue and presenteeism of EMT members in Noto peninsula earthquake in Japan (2024)** **The role of information management and MDS in disaster response** **Predicting the number of consultations by EMTs during disasters using a new statistical model** **Comparative analysis of information management practices by WHO EMTCC during major disasters: Cyclone Idai (2019), Republic of Moldova refugee crisis (2022), Türkiye earthquake (2023), and Palestine humanitarian crisis (2024)** **Information management and action plan determination by national EMT, Japan's DMAT: strategic approaches at the prefectural emergency operation centre** **Strengthen data management capacities of EMTCCs** **Application of advanced technologies by EMTs and EMTCCs in disaster settings: a scoping review**
**New Zealand**	**Sustainable strategies and financial models for EMTs** **New challenges: modularization and implementation strategies** **Future standards: telehealth for health emergency response – partnering in the Pacific: PACMAT experience** **Stopping the bleed: can just-in-time training improve the tourniquet application competencies of bystanders and first responders? A randomized control trial**
**Northern Mariana Islands, Commonwealth of the (USA)**	**Climate change and climate-related emergencies**
**Palau**	**EMT training and capacity-building**
**Papua New Guinea**	**EMT-ECO: synergies with emergency care – the PNG EMT: strengthening standardized emergency care while responding**
**Philippines**	**Climate change and health high-level strategic discussion** **Enabling EMT 2030 implementation** **Global Health Emergency Corps** **Sustainable strategies and financial models for EMTs** **Nonlinear progression and multivariant consideration towards classification and reclassification** **Towards a One EMT information management system** **PEMAT information systems: digitalizing health emergency and disaster response** **PEMAT: what began as a domestic necessity has evolved into a global asset** **Enhancing mass casualty preparedness: inter-agency collaboration in an airport emergency response exercise**
**Republic of Korea**	**Enhancing KDRT’s training and capacity building programs: a systematic review** **Multidimensional analysis of attacks on medical facilities and hospitals: evidence of humanitarian crisis during the Syrian war** **Enhancing KDRT's administration and organizational management through SWOT analysis: a systematic review** **Developing guidelines to enhance technological interoperability of communication and information management systems for KDRT medical teams: a systematic literature review**
**Samoa**	**Highly infectious diseases**
**Singapore**	**Nonlinear progression and multivariant consideration towards classification and reclassification** **A unified government–private sector strategy for streamlined Singapore EMT development**
**Vanuatu**	**EMT deployment in response to Cyclones Judy and Kevin in Vanuatu: coordination, challenges and outcomes**
**WHO Western Pacific Regional Office**	**New standards: implementation strategies and challenges** **Implementation tools for medical care in semi- and non-permissive environments** **EMT warehousing solutions in the Pacific island countries and areas: addressing system and infrastructure challenges to enable emergency deployments** **WHO guidance on research methods for health emergency and disaster risk management: a tool to build and use evidence to protect health and strengthen EMTs** **Progress in health data collection and management during and after emergencies and disasters: increasing evidence by EMT minimum data set** **Designing an EMT cache for extreme cold weather in Mongolia**

AUSMAT: Australian Medical Assistance Team; DMAT: Disaster Medical Assistance Team; ECO: emergency, critical and operative care; EMT: emergency medical team; EMTCC: emergency medical team coordination cell; FEMAT: Fiji Emergency Medical Assistance Team; FETP: field epidemiology training programme; KDRT: Korea Disaster Response Team; MDS: minimum data set; PACMAT: Pasifika Medical Association Medical Assistance Team; PEMAT: Philippine Emergency Medical Assistance Team; PHRRT: public health rapid response team; PNG: Papua New Guinea; SWOT: strengths, weaknesses, opportunities and threats; WHO: World Health Organization.

Over 100 governmental and nongovernmental EMT representatives from more than 21 countries and areas attended the meeting’s regional session, which included the nomination of a new Regional Chair Group and a “world café” session to engage participants in shaping the future regional response. The Western Pacific Regional Group nominated and selected the new Regional Chair Group: Incoming Chair – Philippines; Outgoing Vice-Chair – Samoa; Incoming Vice-Chair – Papua New Guinea; and Nongovernmental Representative – Pasifika Medical Association.

The world café session focused on eight thematic topics covering national, regional and international deployments, interoperability, EMT logistics, water, sanitation and hygiene (WASH), specialization, training and the impacts of climate change on emergency response. Key takeaways from the regional session emphasized the need to expand collaborative training, including topic-specific training and exercises for EMT logistics, WASH, sustainable practices, EMT coordination and climate change actions. Participants from the Region endorsed the creation of an online platform for EMTs and members to mainstream collaboration and sharing. They emphasized the need to continue strengthening interoperability, national EMTs, national EMT governance, and subregionalization with a common training content, common cache, regular joint exercises and common information management systems.

The EMT Global Meeting served as a valuable platform for teams to share operational experiences and engage in strategic discussions on the future growth of the EMT initiative, (*4*) which aims to enhance the speed and quality of national and international EMT responses. Dialogue from the meeting will help shape regional priorities for health emergency preparedness and response moving forward. A key benefit of such gatherings is the opportunity for participants to build relationships and trust before collaborating during a health emergency response. The meeting highlighted the successes in the Western Pacific Region and continued progress towards a more mature, integrated approach to emergency medical response.
